# The Influence of Sustainability on Identities and Seafood Consumption: Implications for Food Systems Education for Generation Z

**DOI:** 10.3390/foods12101933

**Published:** 2023-05-09

**Authors:** Kristin E. Gibson, Catherine E. Sanders, Allison R. Byrd, Kevan W. Lamm, Alexa J. Lamm

**Affiliations:** 1 Department of Agricultural Leadership, Education, and Communication, College of Agricultural and Environmental Sciences, University of Georgia, Athens, GA 30602, USA; 2 Department of Agricultural and Human Sciences, College of Agriculture and Life Sciences, North Carolina State University, Raleigh, NC 27695, USA

**Keywords:** family identity, Generation Z, place attachment, seafood consumption, sustainable seafood

## Abstract

Seafood is a vital source of nutrition yet many consumers in the United States have been exposed to competing discourse about the industry’s environmental impacts, influencing consumption habits. Generation Z, a generational cohort whose members value the sustainability of their purchasing decisions, may have unique opinions regarding sustainable seafood given their sustainability values. This qualitative study explored Generation Z undergraduate students’ experiences with seafood and how they perceive the role of seafood in feeding people while sustaining the future natural environment. Data were collected using 11 focus groups in undergraduate classrooms. Researchers conducted an emergent thematic analysis and sufficient interrater reliability was established. Themes identified based on participants’ experience with seafood included geographic location, experience fishing or with fishermen, and seafood and family, implying place attachment and family identity were intertwined with consumption behaviors. Themes identified based on participants’ perception of seafood’s role in feeding people included sustainability, regulations, limited seafood consumption, and limited knowledge, implying Generation Z’s emerging status as the sustainability generation. Results indicate educators should focus on how sustainability can be emphasized in the classroom with clear actions undergraduate Generation Z students can take to improve sustainability.

## 1. Introduction

Blue food systems are at the forefront of global conversations as the United Nations (UN) strives to meet its Sustainable Development Goals (SDGs) [1]. Historically, food security discussions centered around land-based agriculture, overlooking the role of seafood in the food system [1]. Yet, more than three billion global consumers use blue foods as a vital source of nutrition and many rely on blue food systems to support their livelihood [1,2]. The first UN Food Systems Summit, which convened in September 2021, encouraged discourse surrounding blue food systems and spurred global actions with measurable outcomes to achieve the SDGs while recognizing the complexities of food systems [1]. Sustainable blue food systems—and sustainable fishing practices needed to ensure a viable long-term food source while caring for the natural environment—will play an integral role in meeting future food security [1].

There is a lack of consensus, however, on what sustainable fishing means within the scientific community [3]. There are complex models of population dynamics for sustaining fish species but sustainability questions arise regarding human versus non-human food needs and other ecological considerations [3]. Human values alter the definition of sustainability, causing it to vary based on region and time; therefore, it cannot be reduced into a singular definition [3,4]. Yet, news stories and campaigns in the mid-1990s initiated a sustainable seafood movement that paralleled ethical consumerism and established a foundation for consumers’ knowledge of what is considered sustainable seafood [5,6]. The sustainable seafood movement caused a myriad of organizations, ranging from corporations such as Walmart to non-governmental organizations (NGOs) [7,8], to create certifications, standards, and best management practices [5] further impacting consumers’ knowledge of what is considered sustainable seafood. However, consumers were faced with competing discourse. For example, some entities encouraged seafood consumption for health benefits while others emphasized the negative environmental impacts of increased seafood consumption. Uncertainty around safe seafood consumption resulted from these conflicting messages [9]. Literature about seafood consumption generally focuses on taste, health benefits, and diet variety [10]. Environmental impacts, sustainability, and the consumption of local food have more recently spurred discourse in the seafood consumption literature [9].

Generation Z, a generational cohort in the United States (U.S.) whose members value the sustainability and environmental impact of their purchasing decisions, will soon play a significant role in the marketplace with their purchasing power [11]. A typical member of Generation Z was born after 1996 and is a digital native, meaning they grew up with technology and have an innate literacy and understanding of how to navigate technological developments [12,13]. Although there is no exact definition for generational cutoffs, journalism and popular culture spurred momentum for the name Generation Z in 2019 and the name is well accepted by research organizations and dictionaries [13]. Generation Z is preceded by the Millennial generation (born between 1981 and 1996) who is technologically savvy but adapted to technology unlike a digital native [13,14]. Generation Z has a similar perspective to Millennials on an array of environmental issues, but most Millennials joined the workforce during an economic recession, shaping their adulthood and behavioral patterns [13,15] There are strong generational divides between Generation Z and Generation X (born between 1965 and 1980) and Baby Boomers (born between 1946 and 1964) who are more resistant to environmental protection—such as climate change initiatives [15]—and did not grow up with constant connectivity to the world [13,14]. Generation Z has more options, including seafood options [16], in the marketplace when compared to generations before them because of the emergence of the global market and the internet [17], which may impact how they view and purchase seafood.

Achieving sustainable food systems is a complex challenge requiring both upstream efforts such as structural and cultural changes and downstream efforts such as green consumption, or the consumption of products that safeguard the environment [18]. However, consumers are often unwilling to engage in sustainable consumption practices as they frequently require reducing the convenience of previous shopping habits [18,19]. Considering this, Generation Z may have unique opinions regarding sustainable seafood given their sustainability values. The influence of these values on purchasing habits and the marketplace in the future is anticipated [11]. Many Generation Z consumers are still in school, including the undergraduate and graduate level, suggesting classroom education about seafood can still reach Generation Z. Therefore, it is important to explore the experiences of Generation Z in the undergraduate classroom with seafood and how they perceive the role of seafood in feeding people while sustaining the natural environment. Educating Generation Z may be one solution for creating a future workforce prepared to address complex problems in the food systems from a holistic, socio-ecological perspective [20].

### 1.1. Consumption of Seafood

Factors which influence food consumption behavior are complex as they extend beyond hunger or nutrition into personal and socio-cultural dimensions [21,22]. Previous studies have found numerous factors influence the consumption of seafood [23], such as sensory attributes such as freshness or taste [9,24], consumption during childhood [9,10,25], regional variability or place [26], and demographics [27]. Birch and Lawley (2013) examined if consumption habits, childhood seafood consumption, seafood familiarity (purchasing, storing, preparing, and serving experience), and attitudes towards seafood influenced Australian consumers’ consumption of seafood [10]. The results indicated regular seafood consumption during childhood had a positive relationship with respondents’ attitudes towards seafood and familiarity with seafood as an adult. In addition, respondents who consumed seafood regularly held more favorable attitudes towards seafood and were familiar with seafood in general [10].

Murray et al. (2017) explored factors important to grocery store consumers’ seafood purchasing habits and variables with the potential to predict those habits in three coastal communities in British Columbia [9]. The results indicated seafood sensory attributes, such as taste, smell, and appearance, were the most important factors in grocery store consumers’ seafood purchasing habits. Seafood consumption during childhood also had a positive relationship with respondents’ adult consumption frequencies. In addition, the provenance of the product, such as local and non-local products or farmed versus wild, was important to grocery store consumers’ seafood purchasing habits. However, the perceived sustainability of the species was not found to be an important factor in seafood purchasing habits [9].

Cardoso et al. (2013) investigated gender and regional variables with seafood consumption preferences, consumption frequencies, average meal portions, and culinary treatments in Portugal, which has the largest per capita seafood consumption of countries in the UN [26]. The results indicated regional variability, mostly distance from the sea, played a role in fish consumption. For example, respondents from coastal communities preferred wild fish. Cardoso et al. (2013) noted, however, cultural factors also influenced seafood consumption in Portugal and, therefore, results may vary elsewhere [26].

Scant literature exists informing the background motivations and habits influencing U.S. seafood consumption. The U.S. Department of Health and Human Services recommendations include consuming two servings of seafood per week, and the weekly consumption of eight ounces of seafood is associated with reduced chances of cardiac death [27]. Despite these guidelines, from 2013 to 2016, only 20% of U.S. adults ages 20 and up consumed seafood at least twice per week. Additionally, weekly seafood consumption habits varied significantly based on race and ethnicity. Forty percent of adults who were non-Hispanic Asian consumed seafood at least twice per week, almost double the seafood consumption of “non-Hispanic white (18.7%), non-Hispanic black (22.6%), and Hispanic (14.5%) adults” [27] (p. 2). Only 6% of youth between ages two and 19 consumed seafood at least twice per week, whereas 20% of non-Hispanic Asian youth reported these consumption habits. Additionally, seafood consumption decreased for both adults and youth in the U.S. from 2005 to 2016 [27]. Examination of the personal factors behind seafood consumption habits, especially within groups of younger U.S. consumers, could be beneficial to understanding how to integrate more sustainable seafood consumption into the U.S. public diet.

### 1.2. Place Attachment and Family Identity

Identity is interconnected with food consumption in Western societies [19,28,29,30]. Consumers’ awareness of the environmental impact of their food choices is increasing, resulting in identity shifts that influence consumer behavior [31]. Social interactions and expectations based on different contexts influence consumption behavior [32], including place and family context.

While sustainable behaviors and patterns of consumption are often explored using identity [11,19,31,33], there is limited research on the effect of place identity on sustainable consumption [34]. According to Lee et al. (2015), residents who have a strong sense of place identity, associated with valuing a clean and healthy environment, have more positive attitudes toward sustainable consumption [34]. Place identity encompasses levels of attachment to a physical residential environment, continuity with and experiences of personal past in relation to the environment, and levels of familiarity, cohesion, and social acceptance from environmental and social feedback [35]. Place identity is influenced by an individual’s beliefs, feelings, and values in relation to one’s physical environment and defines one’s self-concept in relation to the environment in which they are situated [36,37]. Belongingness influences one’s place attachment to a physical location mediated by community interactions, through “a deep sense of being an integral part of one’s community [..and] a feeling that one’s values and worldview are natural and right because they are essentially the same as those held by the rest of one’s community” [38] (p. 96). When looking at consumption patterns related to seafood, assessing consumers’ place identities in relation to the product may prove valuable, as “efforts to encourage a more positive attitude to sustainable consumption can be enhanced by strengthening the ties between individuals and their communities” [35] (p. 587). Thus, if a consumer has a place attachment to a location associated with fishing or seafood, they may be more apt to support policies that enhance the social, economic, and environmental sustainability of the industry [35].

Place attachment also connects to familial identity, as family attachment is a key component of why individuals choose to stay in a place, neighborhood, or community [39]. Affective connections, such as familial ties and family membership, are core components of identity [40,41]. Raymond et al. (2010) found that family bonding was a predictive variable for place attachment, connecting physical spaces with personal emotions formed through social connections [42]. Looking at people’s connection with the seafood industry, and experience with fishing both through place attachment and family identity may provide further insights into the relationships between social experiences and consumption patterns. Social norms and emotional attachment both play a factor in engaging in fishing as a hobby, where it can serve as “a valuable opportunity for quality family time” [43] (p. 118), as well as influencing food preferences through social norms and family environment [44].

Few U.S.-based studies have explored the consumption of seafood and its perceived sustainability as part of the food system (e.g., [24]), yet seafood is considered an important component of future food security [1]. Generation Z will soon have a strong influence on the marketplace [11], making their opinions regarding seafood particularly pertinent to food security discussions. It is important to understand Generation Zs’ experiences with place attachment and family identity while growing up in a digital world that exposed them to information inaccessible to previous generations as it may influence the impact of place attachment and family identity on food consumption. Therefore, this qualitative study sought to develop a rich understanding of Generation Z students’ experiences with seafood and how they perceive the role of seafood in feeding people while sustaining the future natural environment. Two research questions guided the study:

1. How do undergraduate Generation Z students describe their experience with seafood?

2. How do undergraduate Generation Z students conceptualize the future of seafood’s role in feeding people while also sustaining the natural environment?

## 2. Materials and Methods

### 2.1. Participants

The population of interest for the current study were Generation Z consumers in the U.S. A convenience sample was used to recruit participants who represented a small portion of this sample, specifically, undergraduate students at the University of Georgia, a land-grant university in the southeastern U.S. [45]. Georgia has a strong culture of land-based agricultural production and a coastline that contributes to the seafood market, making it an appropriate location for the study considering participants have experiences with both land-based and blue food systems. Potential participants were recruited from courses taught in the College of Agricultural and Environmental Sciences between November 2021 and April 2022. Courses, from which students were invited to participate, included an agricultural leadership course, a service-learning course, a floriculture course, and a global food policy course. A range of courses was included to ensure representation from students across the college were present in the sample. Five course instructors allowed the researchers to conduct focus groups during class time, resulting in a convenience sample from researcher and collegiate networks [45]. A total of 68 students representative of the population of interest participated in 11 focus groups. Focus groups ranged in size from two to 11 participants, with an average of six participants. Focus groups were conducted during regularly scheduled class times. Course instructors recruited students to participate in the study; however, no incentives for participation were provided.

The study was approved by the University of Georgia IRB (Protocol #00004479). The instructor of each course advised the students prior to the specified day of the focus group that participation was voluntary. In addition, students were reminded the study was voluntary per IRB protocol when they arrived at class. Students were not penalized for choosing not to participate.

### 2.2. Data Collection

A focus group is a “carefully planned group meeting designed to collect perceptions and information on a defined area of interest” [46] (p. 1). Focus groups are similar to a group interview where questions about a topic are prompted by a researcher (the moderator) and group members discuss that particular topic [47]). Focus groups are particularly useful when the researcher is interested in a group’s shared understanding of a topic and how individual members of the group are influenced by the larger group, a phenomenon influenced by the theoretical perspective of social constructivism [48]. Focus groups are helpful when there is little information available about a particular issue [47], such as sustainable seafood consumption, and the researcher wants to understand perspectives and interpret behavior [47]. The experiences and language of participants create theory during focus groups [49,50].

The epistemological orientation underlying the current study was social constructivism, which defines bodies of knowledge as social constructs emergent from human history and social interaction [48]. From a constructivist perspective, a researcher’s identity cannot be fully removed from the data collection and analysis process, indicating research findings are “created through the researcher’s interaction with the studied phenomenon” [51] (p. 130). Adding another layer of constructed meaning from a social perspective comes from the data collection method itself: focus groups. Focus groups are based on social constructivism as the researcher assumes attitudes, beliefs, and actions are a social process where people form their own opinions through social interactions [47]. Social constructivism considers knowledge and meaning-making as a product of human relationships [52], and thus highlights the data from an interactionist perspective, contrary to an interview where no interaction between participants occurs. Within social constructivism, participants develop subjective and collective meaning from their experiences, and research highlights interactions among individuals in the research situation [53]. Focus groups were selected as the data collection methodology to observe both individual and collective concepts of identity articulation among participants.

A moderator guide was developed to allow participants to discuss their experience with seafood and thoughts about the role of seafood in feeding people while also sustaining the natural environment. An operational definition of seafood was not provided to students; therefore, there was no formal differentiation between wild/farm-raised seafood and salt/freshwater seafood in the prompts. The lack of operational definition should be considered a limitation of the study as students may have different backgrounds which influence what they personally consider when thinking of seafood, ultimately influencing their responses.

At the start of each class, a brief overview of what to expect in the sessions was described by a moderator. For classes with multiple focus groups, participating students were randomly assigned to a group when they entered the classroom. To begin the focus group the moderator first provided a brief overview of the focus group, including best practices for engaging in discussion. Subsequently, the moderator asked participants about their experience with seafood. Depending upon the responses, the moderator asked probing questions such as to what extent participants grew up eating seafood, if participants grew up near the coast or an inland location, and if they knew any fishermen and what their relationship to the fishermen was. Participants were encouraged to discuss any relation or experience they had with seafood. At the end of the focus group, participants were asked to summarize in three sentences or less future needs for the role of seafood in feeding people while also sustaining the natural environment. A panel of experts in agricultural communications, natural resource outreach, and human dimensions of agriculture and the environment reviewed the focus group protocol for face and content validity.

The focus group sessions lasted an average of one hour. Each focus group had one moderator and one notetaker. The same moderator guide was used for all 11 focus groups. Focus groups were conducted in accordance with best practices recommended within the literature [54]. For example, there was minimal input by the moderator throughout the focus groups so conversations flowed naturally and researcher bias was minimized. In addition, the moderator included multiple opportunities for participants to gain insight from all participants throughout the focus group, including allowing all participants to answer in turn two questions, and asking specific individuals for their thoughts if they had not had an opportunity to contribute. The notetaker read a brief summary of participants’ statements at the end of each session for participants to reflect upon and for member checking, in which participants have an opportunity to reflect upon and contribute to the discussion of findings [55].

Trustworthiness is a concept in qualitative research that enhances the credibility, transferability, dependability, and confirmability of findings in relation to the specific sample [55]. Qualitative research engages in a less explicit version of validity, due to the socially constructed nature of the research process [56]. The study’s credibility, or the researchers’ confidence in the accuracy of the findings in relation to the sample, was supported through member checking and methodological triangulation (see [57]). The transferability of the study, defined as the applicability of findings in other contexts, was established through a thick description of the study population and educational context [55]. The study’s dependability was established through an audit trail of data transcripts, researcher coding, and a codebook [58]. Finally, the confirmability of the study, defined as the extent to which findings reflect the intentions and meanings of the participants rather than researcher bias, was supported through the audit trail, researcher reflexivity and peer-debriefing, and data and methodological triangulation (see [57]). All methods of establishing the trustworthiness of the data were done in accordance with recommendations from Lincoln and Guba (1985) [55].

### 2.3. Methodological Limitations

There are several methodological limitations that need to be addressed. First, focus group research is open-ended and, therefore, data produced cannot be predetermined as participants are encouraged to have conversations with each other, ask questions, and express their opinions [50]. Moreover, socially acceptable opinions tend to emerge from focus groups [50]. Next, focus group participants are expressing their views in a particular context and culture and the researcher may not be able to correctly interpret their message [50]. Therefore, focus groups need to be analyzed as a controlled group discussion rather than a discussion that occurs naturally [50]. Next, qualitative research and focus group data is not generalizable [47]. In this sense, focus group results from the current study are limited to the study sample.

### 2.4. Data Analysis

Focus groups were audio recorded, transcribed verbatim, and checked for accuracy prior to analysis. Two researchers conducted a thematic analysis using MAXQDA software to generate codes aggregated to explain patterns identified in the data [53]. Thematic analysis is a multi-step process where textual data are given codes based on shared characteristics and patterns and eventually assigned to larger themes derived from the data itself [58]. According to Braun and Clark (2006), themes are “something important about the data in relation to the research question, and represents some level of patterned response or meaning within the data set” [59] (p. 82). The analysis was emergent because codes and themes were not determined prior to analysis. One focus group was open-coded by two authors to assess interrater reliability to enhance the trustworthiness of the data analysis [60]. Interrater reliability was calculated with Cohen’s kappa and found to be κ = 0.85, which was deemed adequate to move forward with individual coding [61]. Additionally, a codebook was developed through the thematic analysis process as an audit trail and peer debrief to further enhance the trustworthiness of the data [55]. To enhance the transferability of the study [55], a thick description of participants and the research context was provided in previous and subsequent sections. Due to the large number of participants, quotes were attributed to the focus groups to represent the voices present in the social construction of meaning created within each focus group, rather than to individual members of the focus group. For example, quotations pulled from the fourth focus group (FG) are labeled FG 4. Details about each focus group are presented in Table 1.

### 2.5. Data Collection Contex

This study was part of a larger research effort that examined systems thinking in relation to seafood. The overarching study included a quantitative survey and focus group where students addressed hypothetical case scenarios about seafood (see [57]). In addition, a survey was used to collect data on students’ system thinking capacities and green consumerism. Additionally, 68 students participated in the focus groups, with some students on the online synchronous video platform Zoom but most students in person, which may have influenced the flow of conversation and the levels of engagement from in-person compared to online participants. The sample size was deemed acceptable for the scope of the present study as saturation was reached in our sample audience [47,62]. This context is presented in accordance with recommendations within the literature [63].

### 2.6. Participant Demographics

Participant demographics were collected as part of the overall study design. Table 2 presents the demographic characteristics of the participants in the study.

## 3. Results

### 3.1. Students’ Experience with Seafood

Themes were identified based on participants’ experience with seafood. They included geographic location, experience fishing or with fishermen, and seafood and family. Table 3 provides a brief overview of topic areas, corresponding themes and descriptions, the number of codes associated with each theme, and the number of focus groups in which the theme was discussed. Each theme is discussed in detail in the following sections.

#### 3.1.1. Geographic Location

Geographic location impacted participants’ experience with seafood. Many participants preferred eating seafood when they were visiting a coastal community on vacation. One participant considered eating seafood a special and place-based event and said, “[m]y family and myself also predominantly only eat seafood when we go to the coast as a special thing. It’s not a regular part of our diet” (FG 11). Participants also expressed distrust in seafood that was sold inland. For example, one participant said,

“I feel like most of my experience with seafood has been when we’ve taken vacations to the beach […] I feel like that’s where I trust it the most. Whereas at home, I know it doesn’t come locally as much. Maybe it does from aquaculture and stuff like that, but I don’t feel as comfortable eating it as I would in a beach setting or a coastal setting” (FG 8).

Another participant explained, “I personally like seafood when I go to Florida, but I’m scared of eating it here because I don’t really know where it comes from, so I don’t trust it” (FG 6).

Freshness was also expressed as a reason for eating seafood when visiting the coast. One participant said they “only like seafood when I go down to the beach because I just know it’s going to be fresher down there” (FG 3).

Participants who grew up in coastal areas also had a preference for seafood. One participant explained, “I grew up only about 30 to 45 min from the coast. I’ve always enjoyed seafood. We really ate it a lot growing up” (FG 11). Another participant who grew up near the beach said,

“Growing up we weren’t that far from the beach, maybe an hour or so from Florida. We always ate seafood. A big thing that we do with our family is every time we had our families over, we’d have a low country boil with a lot of seafood. It was a pretty big part of my diet” (FG 11).

#### 3.1.2. Experience Fishing or with Fishermen

Experience fishing or with fishermen impacted students’ experience with seafood. Participant experience fishing on their own or with others and/or knew someone who fished, such as a family friend or commercial fisherman, influenced perceptions. For example, one participant explained, “[w]e grew up going deep sea fishing pretty often in the summers and spring break down in Florida, just all off the Atlantic coast and in the Gulf. So, [I] love seafood—always have probably always will” (FG 10). Consumption of fish caught in saltwater was more popular among participants than consumption of fish caught in freshwater. One participant explained,

“We have a lot of lakes in the Midwest, so a lot of my friends hunt and they fish, but they fish for recreation, not really the consumption, just because the fish that you catch on the manmade lakes in Iowa are not very good. So, I know a lot of fishermen, but not in the sense that we eat the food that they catch” (FG 10).

Participants who grew up in inland areas generally did not have experience fishing or with fishermen.

Very few participants knew a commercial fisherman. For example, one participant explained, “I don’t know any fishermen that do it as like a career, but pretty much everyone I know fishes” (FG 9). Another participant had a commercial fisherman in their family and said, “I’ve got family in Florida as commercial fishermen. We go down and fish with them and get both sides of eating it and also catching it” (FG 11).

#### 3.1.3. Seafood and Family

Another identified theme was how familial interactions influenced participants’ experience with seafood. Participants who indicated they currently ate seafood often had seafood with their family growing up, whereas participants who indicated they currently did not eat seafood did not have seafood with their family growing up. One participant said, “[m]y parents made it a lot growing up so it’s a staple food in my diet now, especially salmon” (FG 6). Another participant said,

“My mom is allergic to shellfish, so seafood. We don’t really eat it in my household very much, but my dad and brother eat it. So, I just kind of grew up never eating it and never really being around it because of my mom. So I never really got exposed to it. I’ve tried it now, but my taste palette, I think it is steady in not liking seafood” (FG 4).

One student discussed their family culture in relation to seafood consumption and said “I like seafood but I would describe myself as distant […] because my parents are foreign. My mom is Polish and my dad is Nigerian so I feel like that’s not really our culture to have a seafood boil” (FG 2).

Seafood and family were often associated with geographic location and experience fishing or with fishermen. For example, many participants went on vacation with their families to a coastal area where they would eat seafood. One participant said, “I used to go to St. Simon’s Island a lot with family and we’d always go to seafood restaurants” (FG 10). Another participant said,

“I grew up for the most part in St. Simon’s. We spent a lot of our time there being my grandparents had a house there. We grew up deep sea fishing and pretty much every time we were in St. Simon’s I was eating seafood” (FG 10).

Another example of seafood and family connected to experience fishing or with fishermen was going on family vacations where fishing played a role in family bonding. One participant explained, “[m]y entire family, we always go deep sea fishing—my dad, my uncle. We all go together deep-sea fishing. Yeah. I think I’ve been going since I was little” (FG 7).

### 3.2. Students’ Conceptualization of Seafood’s Role in Feeding People

The second research question explored participants’ perception of seafood’s role in feeding people. The themes which emerged included sustainability, regulations, limiting seafood consumption, and limited knowledge. Again, Table 3 provides a brief overview of topic areas, corresponding themes and descriptions, the number of codes associated with each theme, and the number of focus groups in which the theme was discussed. Each theme is discussed in detail in the following sections.

#### 3.2.1. Sustainability

Sustainability was a primary theme that emerged in the participants’ conceptualization of seafood’s role in feeding people. Many participants discussed the importance of maintaining healthy fish habitats as a mechanism of sustainability to ensure fish are available as a food source in the future. One participant said, “I think that we should focus on the environmental health of those habitats first. And then in turn, I think that having more fish to fish will come” (FG 6). Another participant explained “I think just focusing on our environmental impact and sustainability first, because if there’s no habitat for marine life to grow, there’s not going to be anything for the future, so just making sure of that” (FG 11).

Participants discussed balancing sustainability and feeding people. One participant explained:

“I feel like with all these situations, it’s a really fine line between feeding the population, but also being environmentally cautious. I feel like going forward just find solutions that cater to both because yes, you have to feed your population, but if you exploit the environment, then, in turn, you’re going to deplete your food source. I think finding solutions that think about long-term effects, but also cater, I guess, to both issues” (FG 5).

In contrast, participants discussed the idea that sustainability should be a “top priority” (FG 2) as there are other food sources people can consume.

#### 3.2.2. Regulations

Regulations were a main theme that emerged in the participants’ conceptualization of seafood’s role in feeding people. Regulations were often discussed as a way to increase sustainability but were coded differently than sustainability as they were explicitly mentioned by the participant. One participant explained:

“Having some sort of regulations on fishing and aquatic life conservation is pretty necessary, but at the same time you still need to have those jobs, and that we were talking about local fishermen. So, […] maybe have more laws and regulations on your massive fishing companies, and give a chance for local fishermen who are actually using it to make a living in those areas” (FG 7).

Although regulations were often posed as a way to increase sustainability, one participant expressed the need for “more people to check up on the regulations” (FG 7) so that they are enforced. Similar to sustainability, four participants expressed the need for a “fine line” (FG 5; FG 8) between sustainable regulations and feeding people. One participant explained “Regulation [is] going to be key. I don’t think you can just ban fishing, but also I don’t think you can just go kill the fish in the sea” (FG 8).

#### 3.2.3. Limiting Seafood Consumption

Limiting seafood consumption was a main identified theme in the participants’ conceptualization of seafood’s role in feeding people. Participants explained seafood was “not really a part of most people’s diets” (FG 7), that it should be “geared towards a luxury [item]” (FG 6), and that it should not be a “main food source” (FG 6). One participant noted the complexity of limiting seafood consumption and said:

“I really feel that in the next couple of years, there should be a push to decrease what I would call harvest fishing, as much as I hate to say that because I really appreciate that industry, but I think that can be supplemented by increases in production of fish or aquaculture just because you can make [it…] a little more sustainable. But it’s really a difficult situation all around, I hate to be the person that actually has to make those choices” (FG 11).

Another participant discussed seafood as an industry that should exist locally and said, “From here, I think we should slowly tone down the seafood industry because there are many other more sustainable resources for food. I think that what industry we should keep should be small local businesses that help support local economies and tourism” (FG 3).

Inland fisheries, aquaculture, and traditional land-based farming were often posed as solutions to limiting coastal or offshore fishing. Similarly, participants believed coastal communities could profit from industries other than fishing.

#### 3.2.4. Limited Knowledge

Participants expressed limited knowledge about seafood’s role in feeding people. This often intertwined with sustainability as participants did not know how to make fishing sustainable. One participant said:

“I don’t really know very much about it, but all my friends I know who do know a lot about sustainability are not very optimistic about sustaining fish, and they don’t eat fish and try to really promote not eating fish. So, I assume it’s a big problem” (FG 9).

Other participants indicated they “didn’t know much about how the seafood industry works” (FG 11). From an economic standpoint, one participant explained “I don’t know enough about [the seafood industry], but I really have never heard of the […] fishing industry being like a huge part of our economy” (FG 2).

## 4. Discussion

Achieving sustainable food systems requires efforts such as green consumption, but many consumers are unwilling to engage in sustainable consumption because it requires personal sacrifice [18,19]. Generation Z consumers are defined by their sustainable values and have the potential to influence the marketplace in the years to come but may have limited experiences with frequent seafood consumption [27]. This study sought to develop our understanding of Generation Z students’ experiences with seafood and how they perceive the role of seafood in feeding people while sustaining the natural environment to provide novel insight into their sustainable values’ impact on seafood consumption. The findings indicated geographic location, seafood and family, and experience fishing or with fishermen were all part of their experience with seafood, implying place attachment and family identity were intertwined with consumption behaviors. Sustainability, regulations, limiting seafood consumption, and limited knowledge were identified as ways participants conceptualized seafood’s role in feeding people. As the sustainability generation, Generation Z is an important consumer segment to analyze from a food-system and seafood consumption perspective given their self-identification and emerging market importance [64].

Geographic location was frequently discussed when participants were asked about their experiences with seafood. Participants who grew up in, or frequently visited, coastal areas often had a positive relationship with seafood and preferred eating seafood while in close proximity to the coast. Some participants expressed distrust for seafood when they were not near the coast. It is possible participants associated the term seafood with saltwater rather than freshwater fish, shellfish, and crustaceans, suggesting additional research is needed to understand if place attachment that influences perceptions of seafood is only present in coastal areas. Participants’ place attachment to the coastal areas they visited or lived near likely fostered a sense of trust for food consumed in those areas; however, place attachment research primarily focuses on residential locations. Few studies have analyzed place attachment with tourists or non-residents (see [65,66]) sometimes referred to as mobile place attachment, indicating a need for future research to explore this intersection. The literature demonstrates the impact of place attachment, memory, and lived experience on behavior towards the environment and food consumption practices [67]. Mobile place attachment [65] may imply experiential learning experiences through field trips are warranted when educating about seafood consumption. Future studies should determine if a sense of place attachment can be built upon through educational field trips to coastal areas and if particular excursions mediate the relationship between place attachment and experiential food-systems education. Integrating experiential education components can enhance individuals’ understanding of and behavior towards the environment [68]; however, baseline assessments of student understanding of and experience with scientific and food-based experiences and concepts is critical for facilitating transformational learning [69].

Participants also discussed the role of the family in their consumption of seafood. Participants who consumed seafood had previous experience eating seafood with family growing up and those who were not exposed to seafood growing up often did not consume seafood currently. One participant explained they never developed a taste palette for seafood because their parents did not eat seafood. The findings aligned with Murray et al. (2017) and Birch and Lawley (2013) who found experiences eating seafood during childhood were positively related to seafood consumption as an adult [9,10]. Murray et al. (2017) found sensory attributes were the most important factor in seafood consumption [9], but it is unclear if positive sensory attributes for seafood consumption develop during childhood. In addition, sensory attributes were described by students as a reason to take geographic regions into account when consuming seafood. The uncertainty warrants future research to determine the relationship between consuming seafood with family growing up and sensory attributes. In this study, family identity played a large role in participants’ consumption of seafood.

There was an overlap between geographic location and seafood and family, indicating identity does play a role in seafood consumption, or lack thereof. Consistently connecting individuals with their food system from an early age may leverage identity and help students be more aware of the sustainability of their consumption habits [34]. Food-system educators are uniquely positioned to engage Generation Z consumers in the sustainable consumption of seafood. Pauley et al. (2019) encouraged the use of curriculum and educational efforts to increase graduates (future workforce members and consumers) who are prepared to address complex problems in the food system [20]. Considering this, food-system educators should consider offering fishing workshops for families with young children where they discuss ecosystems and labels on seafood, go fishing, and learn to cook a species of seafood that is considered sustainable and found in the local supermarkets to encourage the development of seafood as a food source at a young age within the family structure.

The majority of participants, regardless of geographic and cultural differences, indicated the seafood industry needs to be more sustainable. The finding contradicted Murray et al. (2017) who found perceived sustainability of the species was not an important factor in seafood purchasing habits [9]. Generation Z is characterized as being more environmentally conscious and motivated by sustainability than previous generations, which affects their consumption patterns and values [11]. Even members of the Millennial generation are trending toward more sustainable consumption, often due to rising challenges related to social, economic, and environmental issues and experiences navigating adulthood amidst these complexities [70]. Therefore, U.S. undergraduate students may have more sustainable mindsets due to the norms of their generation. Considering this, food-system educators should build upon Generation Z students’ current urgency for sustainability from the industry by focusing on how to increase sustainability as opposed to why sustainability is important. For example, a message targeted at answering why sustainability is important should focus on ways fishing equipment can be improved for sustainability purposes and how to implement policy encouraging the adoption of sustainable fishing equipment would be more effective than a message focused on the importance of sustainable fishing equipment.

Many participants knew sustainability and regulations were important for the future of seafood as a food source, but some did not know how to implement sustainable practices, especially in the context of an industry with which they were unfamiliar. Food-system educators should integrate seafood into their curriculums as many participants expressed limited knowledge about sustainable seafood and the associated industry. Future research should investigate students’ knowledge of sustainable seafood practices and labels on seafood at the supermarket that may inform students’ purchasing decisions. The moderator guide for the focus group did not explicitly ask participants about their knowledge related to the seafood industry, suggesting there is room for additional investigation. Knowledge was discussed by more than one participant in two of the five focus groups coded for knowledge, potentially due to the social nature of focus groups where one individual comment may spur additional thoughts for other group members. Moreover, limited knowledge may have been influenced by the geographical locations in which participants grew up. It is possible seafood was not associated with participants’ daily lives considering most participants only visited the coast periodically so they were never exposed to the seafood industry, which is a more prominent industry in coastal Georgia than near rivers or lakes within Georgia.

Although many participants were not from coastal areas, they expressed a place attachment to the coast due to visiting family who lived in a coastal region or while on vacation. Seafood was often associated with these visits and traditions, which may have positively impacted participants’ interest in sustainability in the seafood industry [34]. However, participants did not express a place attachment to inland areas which involved seafood. Place attachment should be explored further because place attachment may have had more complex effects if participants from coastal states were compared to those from landlocked states. Participants from landlocked states may experience a more prominent fishing industry if they are from towns with economies largely centered around lakes or rivers whereas the prominence of the fishing industry in coastal states is often relegated to specific areas.

The results of this study, however, are preliminary and additional research is needed to determine if Generation Z, broadly speaking, values sustainable consumption outside of the university setting. In addition, future studies should quantitatively examine seafood consumption variables, such as place attachment, family identity, and race, to determine if they are related to sustainable consumption across multiple universities and geographies.

### 4.1. Theoretical Findings

The results from the current study supported previous research indicating family ties and social norms influenced participants’ relationships with, and preferences for, seafood. Several participants detailed how their relationship with seafood was formed through family fishing vacations, supporting findings from Young et al. (2016) who found recreational fishing was related to a sense of community and bonding with family. Additionally, participants explained how the presence of seafood in their adolescent diets (related to family social norms) influenced their perspectives on seafood, which supports Olsen (2004) who explained family consumption patterns are determinants of adolescents’ food preferences, such as seafood. Future research should explore the relationship between place attachment, family connections, and social norms when investigating Generation Z’s connection with the seafood industry, using more targeted moderator questions during focus groups to further illuminate the nuanced components influencing such perspectives. Furthermore, quantitative investigations outlining predictor variables for seafood consumption and perception could build upon foundational explorations of the role of identity in Generation Zs’ seafood consumption patterns.

Although the main focus of the study was on identity, participants used systems thinking vocabulary and concepts when describing the future of seafood’s role in feeding people while also sustaining the natural environment. As described in the methods, participants were introduced to systems thinking scenarios and questions as part of the larger study, which may have altered their responses. However, the very brief introduction to systems thinking may have enabled participants to respond to questions with systems thinking vocabulary suggests introducing systems thinking scenarios may help students think through the complex and interactional dynamics associated with the food system [71].

### 4.2. Limitations and Future Research

The present study presents rich qualitative data to understand the experiences of Generation Z students regarding seafood. However, there are several limitations that must be addressed. As mentioned in the methods, the present study is not generalizable beyond the study sample. The study sample was limited to a specific area and context. It cannot be assumed that Generation Z undergraduates have the same experiences as those who are currently in the workforce and may have not attended higher education. In addition, the University of Georgia is likely to attract a certain type of student that may differ from other universities in Georgia. Future research should expand the study sample to include additional universities.

Although the sample size was deemed acceptable for the scope of the study, participants were enrolled in courses taught in the College of Agricultural and Environmental Sciences, which likely influenced how they perceive the food system as compared to students in another discipline. It is also possible there is a bias for land-based agriculture considering the courses offered in the college are typically land-based whereas degrees in fisheries and aquaculture are offered in a different college. Moreover, the focus group took place as part of the class and students may have felt pressured to behave in a way that they would during their normal class. Future studies should consider sampling multiple colleges at a university to ensure numerous experiences of Generation Z undergraduates are represented.

Next, our study sample was predominantly White and participants were classified as juniors and seniors. A similar study of students in the same college with increased student diversity may influence the study results. For example, students who were classified as first-year students or sophomores may have less biased responses from being enrolled in courses in the college due to fewer experiences in the college.

Lastly, the focus group discussion was preceded by a survey with scenarios related to seafood, questions about systems thinking, and questions about green consumerism. It is possible students’ responses to the focus group questions were influenced by the survey and prior discussion in the focus group. The participants were told that there were no right or wrong answers to the survey to attempt to minimize any bias.

## 5. Conclusions

This study provides valuable insight into the sustainable consumption patterns of Generation Z surrounding seafood. Findings suggest Generation Z wants the seafood industry to be more sustainable. Thus, food-system educators need to focus on how sustainability can be improved and actions students can take to improve sustainability. Additionally, Generation Z needs to be exposed to the seafood industry through family or place in order to increase the likelihood of seafood consumption. Study findings demonstrate a need to have an in-depth understanding of generational experiences when researching consumers’ consumption patterns.

## Figures and Tables

**Table 1:** Characteristics of the Focus Groups.

Course Type	Pseudonym(s)	Number of Participants
Agricultural leadership	FG1, FG2, FG3	4, 4, 2
Service-learning	FG4, FG5, FG6, FG7, FG8, FG9	2, 5, 9, 8, 4, 5
Global food policy	FG10	5
Floriculture	FG11	11

**Table 2:** Demographic Characteristics of Participants (*N* = 68).

	F	%
Gender Identity		
Male	20	29.4
Female	47	69.1
Age		
18	3	4.4
19	15	22.1
20	14	20.6
21	16	23.5
22	13	19.1
23	6	8.8
24	1	1.5
Race/Ethnicity ^a^		
White	55	80.9
Black or African American	8	11.8
Asian	2	2.9
Hispanic or Latino/a/x	4	5.9
Prefer to self-describe (Middle Eastern)	1	1.5
Student Classification		
First-year student	4	5.9
Sophomore	12	17.6
Junior	25	36.8
Senior	24	35.3
Graduate student	1	1.5
Other (Exchange Student)	1	1.5
College Enrollment		
College of Agricultural and Environmental Sciences	44	64.7
College of Arts and Sciences	9	13.2
College of Business	6	8.8
School of Public and International Affairs	2	2.9
College of Engineering	1	1.5
College of Journalism and Mass Communication	1	1.5
College of Education	1	1.5
Undeclared	1	1.5

Note. ^a^ Participants were permitted to select more than one race or ethnicity.

**Table 3:** Description of identified themes.

Topic Area	Themes	Theme Description	Codes Corresponding to the Theme	Focus Groups Corresponding to the Theme
Students’ experience with seafood				
	Geographic location	Growing up near the coast or visiting coastal communities. Growing up in landlocked areas.	44	10
	Experience fishing or with fishermen	Participants who fish personally, knew a commercial fisherman, and/or who had family and friends who fish. Participants who did not have experience fishing or know anyone with fishing experience.	43	7
	Seafood and family	Participants who have family members who eat seafood and/or who grew up eating seafood. Participants who have family members with health restrictions regarding seafood and/or who did not eat seafood growing up.	32	9
Students’ Conceptualization of Seafood’s Role in Feeding People				
	Sustainability	Taking care of the ecosystem/habitat. Preservation of the environment.	40	10
	Regulations	Regulatory actions that encourage sustainability.	21	8
	Limiting seafood consumption	Decreasing the amount of seafood people are consuming.	14	6
	Limited knowledge	Uncertainty about how to implement sustainability in the seafood industry.	9	5

## Data Availability

The data presented in this study are available on request from the corresponding author.
